# Data on environmental analysis of natural antioxidant production from walnut husk by a solar photovoltaic-driven system as a replacement for potentially carcinogenic synthetic antioxidants

**DOI:** 10.1016/j.dib.2019.104933

**Published:** 2019-12-04

**Authors:** Zahra Khounani, Homa Hosseinzadeh-Bandbafha, Abdul-Sattar Nizami, Alawi Sulaiman, Sayed Amir Hossein Goli, Elham Tavassoli-Kafrani, Akram Ghaffari, Mohammad Ali Rajaeifar, Ki-Hyun Kim, Ahmad Farhad Talebi, Mortaza Aghbashlo, Meisam Tabatabaei

**Affiliations:** a Microbial Biotechnology Department, Agricultural Biotechnology Research Institute of Iran (ABRII), Agricultural Research, Education, and Extension Organization (AREEO), Karaj, Iran; b Department of Mechanical Engineering of Agricultural Machinery, Faculty of Agricultural Engineering and Technology, College of Agriculture and Natural Resources, University of Tehran, Karaj, Iran; c Biofuel Research Team (BRTeam), Karaj, Iran; d Center of Excellence in Environmental Studies (CEES), King Abdulaziz University, Jeddah, Saudi Arabia; e Faculty of Plantation and Agrotechnology, Universiti Teknologi MARA (UiTM), 40450 Shah Alam, Selangor, Malaysia; f Department of Food Science and Technology, College of Agriculture, Isfahan University of Technology, Isfahan 84156-83111, Iran; g School of Engineering, Newcastle University, Newcastle Upon Tyne, NE1 7RU, United Kingdom; h Department of Civil and Environmental Engineering, Hanyang University, 222 Wangsimni-Ro, Seoul, 04763, South Korea; i Henan Province Engineering Research Center for Forest Biomass Value-added Products, Henan Agricultural University, Zhengzhou, 450002, China; j Genetic Department, Faculty of Biotechnology, Semnan University, Semnan, 35131-19111, Iran; k Faculty of Mechanical Engineering, Ho Chi Minh City University of Transport, Ho Chi Minh City, Viet Nam

**Keywords:** Biodiesel, Walnut waste valorization, Bio-antioxidant, Life cycle assessment, Propyl gallate, Circular bioeconomy

## Abstract

In order to develop a product sustainably, multiple analyses, including comprehensive environmental assessment, are required. Solar-assisted production of walnut husk methanolic extract (WHME) as a natural antioxidant for biodiesel was scrutinized by using the life cycle assessment (LCA) approach. More specifically, the environmental sustainability of WHME antioxidant was evaluated and compared to that of propyl gallate (PG), the most widely used synthetic biodiesel antioxidant, under two scenarios. Additionally, supplementary files including the inventory data consisting of raw data as well as elementary flows, mid-point, and end-point categories are presented. The analysis of scenarios revealed that the use of the natural antioxidant and the avoidance of the chemical antioxidant in biodiesel fuel could be regarded as an eco-friendly approach substantially enhancing the environmental friendliness of biodiesel in particular in terms of human health. Furthermore, given the waste-oriented nature of WHME, the scenario involved its application could serve as a promising strategy to simultaneously valorize the agro-waste and generate a value-added product; a move toward implementing the circular economy approach in the biodiesel industry.

**Table:** Specifications Table

Subject area	Energy
More specific subject area	Renewable Energy, Sustainability and the Environment
Type of data	CSV
How data was acquired	Raw data were taken from industrial and laboratory data for production of biodiesel and antioxidant and were subsequently converted into environmental impacts using the “IMPACT 2002+” methods available in SimaPro 8.2.3.0 software and EcoInvent database v3.0.
Data format	Raw and analyzed
Parameters for data collection	The consumption of martials and energy as well as their respective emissions to water, air, and soil during biodiesel and antioxidants production.
Description of data collection	Foreground data were obtained directly during biodiesel and antioxidants production while background data were obtained from the Ecoinvent v.3.0 database in SimaPro software.
Data source location	Agricultural Biotechnology Institute of Iran (ABRII) (Karaj, Iran); Biofuel Research Team (BRTeam) (Karaj, Iran); Universiti Teknologi MARA (UiTM) (Shah Alam, Malaysia); Isfahan University of Technology (Isfahan, Iran); Semnan University (Semnan, Iran)
Data accessibility	With the article
Related research article	Z. Khounani, H. Hosseinzadeh Bandbafhab, A. S. Nizami, A. Sulaiman, S.A.H. Goli, E. Tavassoli-Kafrani, A. Ghaffari, M.A. Rajaeifar, K.-H. Kim, A. Farhad Talebi, M. Aghbashlo, Mortaza Tabatabaei, Unlocking the potential of walnut husk extract in the production of waste cooking oil-based biodiesel, Renew. Sustain. Energy Rev. (2020). https://doi.org/10.1016/j.rser.2019.109588.

**Table:** 

**Value of the data** • These data could be of use to researchers performing LCA of biodiesel production from waste cooking oil (more specifically, inventory data and the elementary flows to and from the environment during biodiesel production life cycle) • These data could be of use to researchers performing LCA of agro-wastes valorization into natural biodiesel antioxidants. (More specifically, inventory data and the elementary flows to and from the environment during waste-into-natural antioxidant life cycle). • These data could be of use to researchers performing LCA of integrating renewable energies (e.g., solar photovoltaic energy) and agro-wastes valorization processes to generated value-added products. • These data provide the environmental evaluation of WHME valorized into a natural antioxidant for biodiesel as a replacement to its potentially carcinogenic synthetic counterpart, PG. • The data could be of use to the implementation of the circular economy principles in the biodiesel industry.

## 1 Data

In this data paper, details of life cycle assessment (LCA) of solar photovoltaic-driven production of walnut husk methanolic extract (WHME) as a natural antioxidant for waste cooking oil (WCO) biodiesel is presented. While the data related to LCA of propyl gallate (PG) as a synthetic antioxidant was investigated as control. The present data provide supplementary information to our recently published work [1].

Further to Ref. [1], the supplementary files designated as “Raw data-B100 production.csv”, “Elementary flows-B100 production.csv”, “Characterization-B100 production.csv”, “Damage assessment-B100 production.csv”, and “Weighting-B100 production.csv” show detailed list of the inventory data consisting of raw data as well as the elementary flows to and from the environment, mid-point and end-point categories of the environmental impacts associated with the production of 1 kg WCO by LCA (performed using IMPACT 2002 + method).

Moreover, the supplementary files designated as “Raw data-Walnut husk processing.csv”, “Elementary flows-Walnut husk processing.csv”, “Characterization-Walnut husk processing.csv”, “Damage assessment-Walnut husk processing.csv”, and “Weighting- Walnut husk processing.csv” present the inventory data consisting of raw data and the data related to processing of walnut husk for the production of 5000 mg WHME. Moreover, the supplementary files designated as “Raw data-WHME production.csv”, “Elementary flows-WHME production.csv”, “Characterization-WHME production.csv”, “Damage assessment-WHME production.csv”, and “Weighting-WHME production.csv” include the details of the inventory data consisting of raw data and the data related to the production of 5000 mg WHME.

Finally, the supplementary files designated as “Raw data-PG production.csv”, “Elementary flows- PG production.csv”, “Characterization- PG production.csv”, “Damage assessment- PG production.csv”, and “Weighting- PG production.csv” present the detailed inventory data consisting of raw data as well as the elementary flows to and from the environment, mid-point and end-points categories associated with the production of 250 mg PG.

## 2 Experimental design, materials, and methods

Biodiesel has been widely accepted as a promising alternative to mineral diesel due to its unique environmental advantages, in particular lower exhaust emissions [2,3]. However, its low oxidative stability has introduced many challenges, including deterioration of fuel quality during the long-term storage and the consequent deterioration of fuel combustion performance [4]. In response to this shortcoming, various synthetic antioxidants have been used among which PG, because of its string antioxidant properties, is the most widely used [5]. Nevertheless, the application of PG has been found to be potentially carcinogenic [6], and if not during short- or mid-term, its long-term application could jeopardize the sustainability of the biodiesel industry. In light of that, a widespread search has been on-going striving to introduce safe, natural antioxidants as replacement of PG [7].

Moreover, to further enhance the sustainability of the biodiesel industry, other strategies well aligned with the principles of the circular economy should be taken. Those include the integration of other renewable energy carriers to replace fossil fuels used to power biodiesel plants, the valorization of biodiesel production waste streams (glycerol, wash-water, etc.) into value-added products [[8], [9], [10]], and/or the application of waste-oriented/eco-friendly commodities as a replacement to conventionally used ones such as synthetic antioxidants [11].

Efforts are focused on reducing the environmental impact/health hazards of a product. To determine these environmental impact/health hazards, different methods and tools have been proposed among which LCA has attracted a great deal of attention [12,13]. According to ISO, LCA is a scientific approach evaluating the environmental impacts of a product, process, service, and activity throughout its life cycle from raw materials extraction to manufacturing and use/operation up to its end-of-life (cradle to grave) [14,15]. Given its promising attributes, LCA could be regarded as a key platform capable of assisting with the policy/decision-making process to find best paths and methods well in accordance with the very principles of sustainable development and circular economy [16,17]. It should be noted that LCA also enables the assessment of environmental impacts for comparative and improvement/optimization purposes [18].

Therefore, the inventory data based on the elementary flows, mid-point and end-point categories of the environmental impacts associated with the production of a novel and low-cost waste-oriented natural biodiesel antioxidant, i.e., WHME, by using LCA are provided. Solar photovoltaic energy was used as an integrated part of the natural antioxidant production process. Moreover, these environmental impacts were compared with those of PG as the most widely used synthetic antioxidant in the biodiesel industry. On such basis, two different scenarios were defined:

I. *Scenario one (Sc-1):* Including LCA of the production of 1 kg WCO biodiesel doped with 5000 mg WHME. In this scenario, according to the described method by Jakopič et al. [19], the polyphenolic extract of walnut husks was obtained while the extraction system was equipped with a solar photovoltaic system.
II. *Scenario two (Sc-2):* Including LCA of the production of 1 kg WCO biodiesel doped with 250 mg PG.

It should be noted that the above antioxidant concentrations were based on the minimum requirements of the ASTM D6751 standards for oxidation stability index (OSI), i.e., induction period of 3 h. Accordingly, the induction period of WCO biodiesel was prolonged from 1.2 h to more than 3 h using 5000 mg and 250 mg of WHME and PG, respectively.

LCA was conducted through four different phases: (1) goal and scope definition; (2) life cycle inventory (LCI) analysis; (3) life cycle impact assessment (LCIA); and (4) interpretation [14,20]:

### 2.1 Goal and scope definition

The goal was set to develop the inventory data to and from the environment to investigate the environmental impacts of WHME and PG production as WCO biodiesel antioxidant additive. As established by the ISO [14], the “goal and scope definition” step includes a definition of system boundaries and *functional unit* (FU). FU is a measure of the performance of the functional outputs of the studied system for comparison of several products with each other. The FU selected was “1 kg of WCO biodiesel doped with WHME or PG”.

System boundary delimitates analysis process to the inputs and outputs that should be considered. More specifically, the system boundary for biodiesel production from WCO included purification, and finally, WCO conversion into biodiesel by an alkali-catalyzed transesterification reaction. It should also be noted that in the case of WHME and PG production, the system boundary was expanded from the extraction of the raw materials until the end production step of antioxidants.

### 2.2 Inventory analysis

The data inventory stage is a detailed compilation of material and energy inputs, outputs, and emissions to water, soil, and air, in each step of the system, according to FU [18]. The inventory data was compiled of two groups of data sets. The first group of data was obtained from the *foreground data*, which included type and amount of materials and energy used directly in the production of WCO biodiesel, WHME, and PG as well as the related emissions to water, soil, and air. The second group of data was related to the *background data,* including *upstream* activities involved in the production of materials and energy carriers that were taken from the Ecoinvent database (version 3.0) in SimaPro 8.2.3.0 software. The system boundary for the production of WCO biodiesel included three main steps, i.e., collection and transportation of WCO, WCO refining (purification and esterification), and biodiesel production (transesterification). Water, potassium hydroxide, methanol, purified WCO, transport, and electricity were considered as the materials and energy used directly in the WCO biodiesel production process. The raw data related to the amounts of the listed inputs are provided in the supplementary files designated as “Raw data-B100 production.csv”. Subsequently, these raw data were converted into elementary flows to and from air, water, and soil. These data are listed in the supplementary file designated as “Elementary flows-B100 production.csv”.

Furthermore, the raw data and the elementary flows to and from air, water, and soil for the treatment of wastewater and unreacted WCO associated with the production of 1 kg WCO biodiesel are listed in the supplementary files designated as “Raw data-B100 production.csv” and “Elementary flows -B100 production.csv”, respectively.

It should be noted that in this study, the aim was to evaluate the environmental impacts associated with the use of waste materials *vs.* their virgin counterparts. Hence, the environmental impacts of soybean oil production (0.9 kg) were deducted from the environmental impacts of production of WCO biodiesel (1 kg). Details of the inventory data including the avoided elementary flows to and from the environment as the consequence of avoiding using soybean oil for the production of 1 kg of WCO biodiesel are listed in the supplementary file designated as “Elementary flows-B100 production.csv”.

Moreover, the supplementary file designated as “Raw data-Walnut husk processing.csv” provides the raw data related to the amount of energy use (as kWh photovoltaic electricity consumption) for the production of 5000 mg WHME. The elementary flows to and from environment related to energy consumption in the form of photovoltaic electricity for the production of 5000 mg WHME are listed in the supplementary file designated as “Elementary flows-Walnut husk processing.csv”.

Also, the raw data related to the production of 5000 mg WHME including the amount of solvent (methanol) used, the amount of solvent recovered, as well as the amount of energy used to extract the WHME and the amount energy used for solvent recovery are provided in the supplementary file designated as “Raw data- WHME production.csv”. These raw data with the help of EcoInvent database (version 3.0) and by using the SimaPro software were converted into elementary flows to and from the environment and are presented in the supplementary files designated as “Elementary flows- WHME production.csv”.

It should also be noted that in Scenario 1, using 5000 mg WHME led to avoiding 250 mg PG. Therefore, the inventory data including the elementary flows to and from the environment related to avoiding 250 mg PG, were deducted from inventory data including the elementary flows to and from the environment related to 5000 mg WHME production (supplementary file designated as “Elementary flows- WHME production.csv”).

Finally, the supplementary files as “Raw data- WHME production.csv” presents the raw data related to the production of 250 mg PG including the amounts of materials consumed (i.e., propanol, sulfuric acid, water, diethyl ether, sodium hydrogen carbonate, sodium sulfate, and gallic acid) and energy (electricity) used to generate heat, extract the product, and recover the solvent, as well as the amounts of recovered and unreacted materials (i.e., propanol, sulfuric acid, and diethyl ether). Using Ecoinvent database, these raw data were converted into elementary flows to/and from the environment (supplementary file designated as “Elementary flows-PG production.csv”).

### 2.3 Impact assessment

LCIA using the elementary flows identifies and evaluates the amount and significance of the potential environmental impacts [14]. At first, the elementary flows are allocated to impact categories, and their potential impacts are quantified based on the characterization factors [21]. In the second step, characterized impact categories (mid-points) are combined and are grouped in sets such as human health, ecosystem quality, climate change, and resources depletion.

The impact assessment methodology applied herein was the “IMPACT 2002+” that proposes a feasible implementation of 15 mid-points in a combined approach to form four end-points. All mid-points are presented in units of a reference substance and are related to the above-mentioned four end-points or damage categories [22]. Given the advantage of presenting the results as end-points (damage categories), this type of methodology was used to evaluate the environmental impacts associated with the product life cycle of biodiesel and antioxidants. In better words, because the volume of the inventory data including elementary flows is huge, it is virtually impossible to make decisions accordingly. Therefore, the inventory data were converted into mid-points using IMPACT 2002 + methods the details for the production of 1 kg WCO biodiesel (“Characterization-B100 production.csv”), 5000 mg WHME (“Characterization-Walnut husk processing.csv” and “Characterization-WHME production.csv”), and 250 mg PG (“Characterization-PG production.csv”) are presented. However, there are 15 mid-point categories requiring a high level of expertise to interpret. In light of that, the mid-points were converted into end-points, i.e., ecosystem quality, human health, climate change, and resource damage categories for ease of understanding (See supplementary files designated as “Damage assessment-B100 production.csv”, “Damage assessment-Walnut husk processing.csv”, “Damage assessment-WHME production.csv”, and Damage assessment-PG production.csv”).

The analysis of the damage categories (end-points) for the investigated Scenarios revealed that the application of the solar photovoltaic-extracted natural antioxidant in biodiesel led to approximately 614% lower impacts in resources damage category compared to the application of PG. Furthermore, the use of the natural antioxidant in biodiesel was also environmentally advantageous in the other damage categories *vs.* PG, i.e., 0.32% in ecosystem quality, 12.13% in human health, and 8.37% in climate change damage categories.

Finally, the damage categories (end-points) were weighted to obtain a single score and the results obtained are presented for the production of: 1 kg WCO biodiesel (supplementary file designated as “Weighting-B100 production.csv”), 5000 mg WHME (supplementary files designated as “Weighting-Walnut husk processing.csv” and "Weighting-WHME production.csv”), and 250 mg PG (supplementary file designated as “Weighting-PG production.csv”). The results obtained showed that the single score for Sc- 2 was −587.95 μPt/FU while this stood at −641.35 μPt/FU for Sc- 2. In better words, considering the total environmental damage, Sc- 1 was 9.08% more environmentally friendly than Sc- 2.

## Appendix A Supplementary data

Characterization-B100 production.csv Characterization-B100 production.csv

Characterization- PG production.csv Characterization- PG production.csv

Characterization-WHME production.csv Characterization-WHME production.csv

Characterization-Walnut husk processing.csv Characterization-Walnut husk processing.csv

Damage assessment-B100 production.csv Damage assessment-B100 production.csv

Damage assessment- PG production.csv Damage assessment- PG production.csv

Damage assessment-WHME production.csv Damage assessment-WHME production.csv

Damage assessment-Walnut husk processing.csv Damage assessment-Walnut husk processing.csv

Elementary flows-B100 production.csv Elementary flows-B100 production.csv

Elementary flows- PG production.csv Elementary flows- PG production.csv

Elementary flows-WHME production.csv Elementary flows-WHME production.csv

Elementary flows-Walnut husk processing.csv Elementary flows-Walnut husk processing.csv

Raw data-B100 production.csv Raw data-B100 production.csv

Raw data-PG production.csv Raw data-PG production.csv

Raw data-WHME production.csv Raw data-WHME production.csv

Raw data-Walnut husk processing.csv Multimedia component 16

Weighting-B100 production.csv Weighting-B100 production.csv

Weighting- PG production.csv Weighting- PG production.csv

Weighting-WHME production.csv Weighting-WHME production.csv

Weighting- Walnut husk processing.csv Weighting- Walnut husk processing.csv
